# Single‐center implementation of endoscopic submucosal dissection (ESD) in the colorectum: Low recurrence rate after intention‐to‐treat ESD

**DOI:** 10.1111/den.12995

**Published:** 2018-01-03

**Authors:** Andrej Wagner, Daniel Neureiter, Tobias Kiesslich, Gernot W. Wolkersdörfer, Thomas Pleininger, Christian Mayr, Christiane Dienhart, Naohisa Yahagi, Tsuneo Oyama, Frieder Berr

**Affiliations:** ^1^ Department of Medicine I Paracelsus Medical University Salzburg Austria; ^2^ Institute of Pathology Paracelsus Medical University Salzburg Austria; ^3^ Laboratory for Tumour Biology and Experimental Therapies (TREAT) Institute of Physiology and Pathophysiology Paracelsus Medical University Salzburg Austria; ^4^ Division of Research and Development for Minimally Invasive Treatment Cancer Center Keio University School of Medicine Tokyo Japan; ^5^ Department of Endoscopy Saku Central Hospital Advanced Care Center Nagano Japan

**Keywords:** colorectal neoplasm, endoscopic submucosal dissection, learning curve, postoperative complication, recurrence

## Abstract

**Background and Aim:**

Colorectal endoscopic submucosal dissection (ESD) shows higher R0 resection and lower local recurrence rates than endoscopic mucosal resection (EMR) in Japan. In Europe, independent learning of ESD in the colorectum is feasible, but yet to be analyzed for curative resection and recurrence rates.

**Methods:**

After experimental training under supervision by Japanese experts (T.O., N.Y.), three endoscopists independently carried out 83 ESD procedures intention‐to‐treat for lesions in the entire colorectum of 67 patients in a prospective registry (November 2009 to June 2016).

**Results:**

ESD was feasible in 80 (96%) colorectal neoplasias (mean diameter 33.6 [± 1.8] mm), and three more required conversion to piecemeal EMR. The lesions were adenomas in 66% with low‐grade intraepithelial neoplasia (LGIN), 29% with high‐grade intraepithelial neoplasia, and 5% with carcinomas (G2, pT1). ESD had to be facilitated by the final use of snaring (hybrid‐ESD,* n* = 45), especially in the initial learning period. En‐bloc resection rate was 85%. Complications were microperforations (7%, conducive to one hemicolectomy), and delayed bleeding (1%) without mortality or long‐term morbidity. Residual adenomas with LGIN (5%) after hybrid‐ESD did not recur after endoscopic ablation. All malignant neoplasias (34%) were curatively resected without recurrence after a mean follow up of 19.5 (± 3.2) months.

**Conclusions:**

During independent ESD learning in the colorectum, ESD intention‐to‐treat showed a low recurrence rate after appropriate training, and hybrid‐ESD showed acceptable complication and recurrence rates, justifying hybrid‐ESD as a strategy for self‐completion and rescue.

## Introduction

Endoscopic submucosal dissection (ESD) allows for en‐bloc resection of large superficial pre‐/malignant neoplasias, and aims for curative resection of early gastrointestinal cancer avoiding recurrence or resective surgery. Tutored by experts, ESD has rapidly spread in Japan, but still demands untutored learning in Western countries. A technical step‐up approach that starts with the easiest gastric neoplasias is recommended,^1^ but fails as a result of their low prevalence in Western countries. A prevalence of neoplasia‐driven approach offers an adequate caseload (about two ESD per month), but is based on self‐learning of ESD procedure on more challenging colonic lesions.

However, international results of colorectal ESD are still very heterogeneous:^2^ Asian studies give a benchmark of 93% en‐bloc resection, 86% margin‐negative (R0) resection, 1.1% local recurrence, complications in <7% and secondary surgery in 1% of procedures, respectively. Non‐Asian studies have failed to reach these criteria, and data are very sparse on colonic ESD proximal of the rectum.^2^ These facts question the self‐learning approach in the entire colorectum to implement the ESD technique.

When implementing ESD in a Western referral center, endoscopic electrosurgical competence is the first goal and is defined by a moderate rate of complications (<10%) with a low rate (<5%) of secondary surgery or recurrence.^3^ Subsequent skilled learning aims for a professional level with <5% complication rate and reduced procedure time.^4^ Hybrid‐ESD (H‐ESD) (i.e. snaring of the final submucosal bridge) had been recommended for self‐completion of resection during the learning curve.^5^ Nevertheless, colorectal H‐ESD has been disclaimed as a standard technique because of higher rates of complications and recurrence in a recent meta‐analysis.^2^

For implementation of the ESD technique, we started a prospective case registry of ESD intention‐to‐treat (ESD‐ITT) to analyze the outcomes of resection, rate of complications, procedure‐related morbidity and mortality, need for secondary surgery, and rate and outcomes of recurrent neoplasia or systemic recurrence of malignant disease.

## Methods

Methods and short‐term outcomes of the first 50 independent ESD‐ITT procedures for implementation of the technique have been described in detail.^6^

### Preparation phase

The senior interventional endoscopist (F.B.) had visited Dr Oyama and Dr Yahagi to observe and comprehend 15 procedures in the upper and lower gastrointestinal (GI) tract. Accordingly, F.B. trained in the ESD technique with his team in isolated organs *ex vivo* for 6 months, and organized and participated in an *in vivo* experimental workshop tutored by experts from Japan.^7^ Equipment and strategy improved during his first 25 clinical ESD procedures, and he encountered four microperforations in the colorectum up to procedure no. 23 before achieving a competence level without further complication until procedure no. 50.^6^ Subsequently, he tutored the ESD learning of two experienced interventional endoscopists (A.W., G.W.). Both had previously passed *ex vivo* training, an annual ESD training workshop,^7^ and some of the ESD clinical tutoring events held in Salzburg from November 2011 until February 2015.^8^

### Case series

From November 2009 until June 2016, three gastroenterologists carried out 83 colorectal ESD‐ITT procedures (F.B. *n* = 55; A.W. *n* = 25; G.W. *n* = 3) on 67 consecutive patients (male/female: 47/20; median age 77 years, range 37–93) at an endoscopic referral center (Department of Medicine I, University Hospital, Salzburg, Austria). Additional independent ESD procedures in the stomach (*n* = 9), esophagus (*n* = 4), and duodenum (*n* = 2) were of minor importance for the caseload. Before onset of the clinical tutoring program, 45 untutored ESD procedures (37 colorectal) had been carried out during the learning curve.^6^ Criteria given by Tanaka *et al*.^9^, ^10^ were used to establish the indications, later on confirmed by the Japanese guidelines.^11^ Contraindications were signs of deep submucosal tumor invasion or metastasis, submucosal tumors, and American Society of Anesthesiologists (ASA) status >III. Patients were informed in detail about the ESD procedure, benefits and increased risks of this novel method, necessary endoscopic follow up, and alternative resection techniques (piecemeal endoscopic mucosal resection [EMR] or surgical resection). All patients gave oral and written informed consent for ESD. All data were prospectively recorded for quality control. This consecutive case series was concluded in June 2016; follow up was included until June 2017 for retrospective analysis of outcome parameters. Implementation of established therapies is not subjected to ethics committee approval according to §30 of Salzburg county hospitals act (LGBI nr. 24/2000 and nr. 91/2010).

### Diagnostic work‐up

During endoscopy for ESD indication, all lesions were classified according to Paris‐Japan, and laterally spreading tumor type (LST) classifications, Sano capillary pattern,^12^ and Kudo pit pattern^13^ on magnifying (40× or 100×) endoscopy with white‐light and narrow band imaging using chromoendoscopy with indigocarmine (0.15% aqueous) and/or crystal violet (0.05% aqueous^6^).^14^ On malignant lesions, we examined the echo band of the submucosa layer for integrity with endoscopic ultrasound (20 MHz Fujinon Sonoprobe; Fujifilm, Tokyo, Japan),^15^ excluded enlarged lymph nodes or organ metastasis by computed tomography scan of the abdomen, rectal endosonography (7.5 MHz; Olympus), or chest radiogram. Endoscopic procedures were carried out in unconscious sedation with midazolam and propofol.^6^, ^16^

### Instruments

In the right hemicolon, we used standard or pediatric colonoscopes (CF‐H180 AL, later on CF‐HQ190, PCF 180 AL) with a straight‐shape transparent hood (D‐201‐11804; D‐201‐12704; D‐201‐15004), and gastroscopes in the left hemicolon (GIF‐Q180J, GIF‐HQ190; Olympus Medical Systems, Tokyo, Japan) with CO_2_ insufflation. ESD instruments were dual knife^®^ and/or hook knife^®^, Coagrasper^®^ forceps and, when appropriate, monofil snares (all from Olympus) powered by VIO‐300D electrosurgical generator (ERBE, Tübingen, Germany).^6^

### ESD procedure

**Figure 1:**
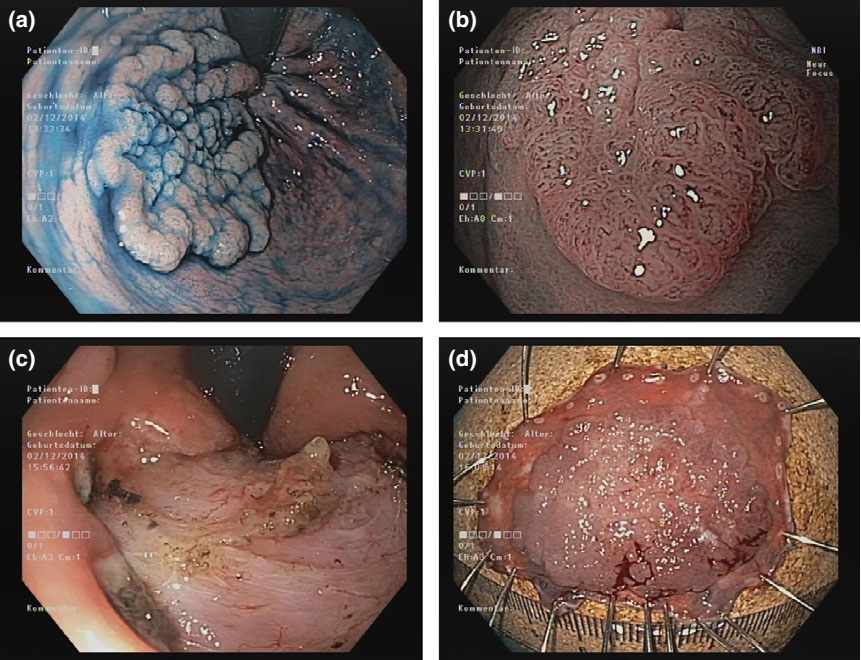
Endoscopic submucosal dissection en‐bloc of a rectal laterally spreading tumor, granular type (tubular adenoma with low‐grade intraepithelial neoplasia). (a) Retroflex view of the tumor approaching the linea dentata after dyeing with indigocarmine, (b) capillary pattern, (c) resection bed, (d) specimen with preserved markings.

Endoscopic submucosal dissection intention‐to‐treat aimed for en‐bloc ESD; when not feasible, additional snaring was used to achieve complete resection. After extensive circumferential submucosal dissection, H‐ESD with up to three pieces was allowed for self‐completion in the case of very long duration or unexpected technical difficulty, and as rescue strategy for complications – or conversion to piecemeal snaring (PM‐EMR, >3 pieces) when the remaining submucosa bridge was still extensive or very fibrotic.^6^ We used 10% glycerol solution (Glyceol^®^; Chugai Pharmaceutical, Tokyo, Japan) for submucosal injection, dual knife for mucosal incision and submucosal dissection,^17^ and hook knife for approach perpendicular to the proper muscle layer^18^ (Fig. 1). Micro‐/perforations were closed with clips (EZ clip; Olympus). Exposed arteries were coagulated and/or clipped.^6^

Specimens pinned on cork board in 4% buffered formaldehyde solution underwent image documentation and histological assessment in 2‐mm‐thick serial sections according to the Vienna and the WHO classification systems.^19^, ^20^ Tumor cell‐free vertical and horizontal margins were classified as R0 resection, margins with micro‐focal residual adenoma as R1, and resection in more than one piece as Rx. Resection R0 was classified “curative” when carcinoma grading was G1 or G2, in the absence of deep submucosal invasion (>1000 μm depth), tumor cell budding, and lymphatic or vascular invasion (on immunohistochemistry for podoplanin or CD34).^21^

### Definition and management of complications

Any perforation (i.e. mural tissue defect with visible peritoneal cavity or retroperitoneal fat) was immediately closed with clipping, followed by i.v. antibiotics, parenteral nutrition (for ≥24 h), and in‐hospital follow up for at least 2 days.^10^, ^22^, ^23^ Delayed bleeding was defined as bloody stool and need for endoscopic hemostasis after ESD.

### Follow‐up examinations

Post‐ESD in‐hospital surveillance included serum laboratory testing and clinical follow up (at 6–8 and 24 h). Follow‐up colonoscopy was scheduled at 3–6 months, and later on according to neoplasia (adenoma/high‐grade intraepithelial neoplasia [HGIN]/cancer pT1a/pT1b) and resection status (R1/R0) at 1 to 3 years after ESD, as in current guidelines.^20^

### Statistics

Data were summarized as median and range or mean values with standard error of the mean (SEM), as appropriate. For significance of comparison, we used Pearson's chi‐squared test for categorical variables, and Mann–Whitney's *U*‐test for numerical variables. Binary logistic regression was applied to explore for predictors of technical performance and outcome during ESD implementation by three operators, using rates of complications, en‐bloc resection rates, R0 status and recurrence as the dependent variables. *P*‐values <0.05 were considered statistically significant. Analyses were carried out using the statistical package SPSS version 17.0 (SPSS Inc. Released 2008. SPSS Statistics for Windows, Version 17.0. SPSS Inc., Chicago, IL, USA).

## Results

### Lesion characteristics

**Figure 2:**
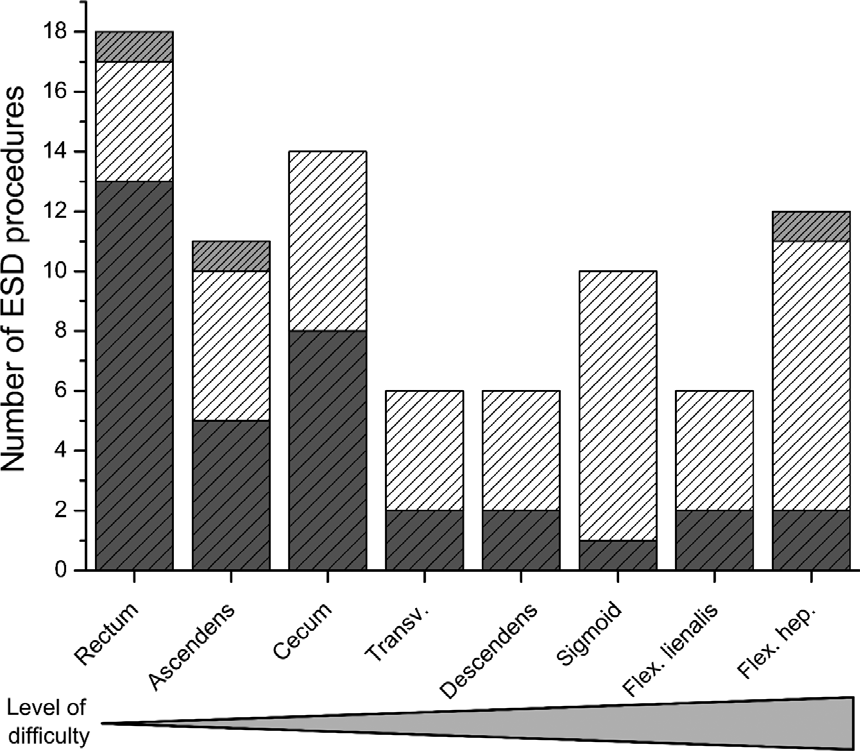
Number of endoscopic submucosal dissection (ESD) procedures (*n* = 83) listed according to technical difficulty of anatomical location,^30^ with associated number of (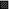) ESD, (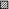) hybrid‐ESD (H‐ESD), and (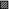) piecemeal resections (PM).

Altogether, 83 consecutive lesions (diameter median 30 mm, range 15–80 mm) fulfilled indication criteria for colorectal ESD.^10^ In the study period, we had observed only three LST with endoscopic signs of submucosal invasiveness (exclusion criteria) and non‐lifting on submucosal injection, then referred for surgery (all were pT1b or T2 cancers). Half of the neoplasias (49%) had a non‐granular, and 0‐IIa, 0‐IIa+IIc, or 0‐IIc type morphology. The largest lesions (*d* >50 mm) were LST granular mixed (LST‐GM) type. The lesions comprised 55 adenomas with low‐grade intraepithelial neoplasia (LGIN, 66%) and 28 malignant lesions (34%); i.e. 24 (29%) adenomas with HGIN and four (5%) early low‐risk carcinomas (Tis [M], *n* = 3 and T1a [SM], *n* = 1, all graded G2). Malignant histopathology (HGIN or carcinoma) mainly occurred in pseudo‐depressed lesions or LST‐GM type (Table 1). Most neoplasias (78%) were located proximal to the rectum including all difficult locations for ESD (Fig. 2). Eighteen neoplasias (22%) were at colonic flexures, and 22 (27%) showed severe submucosal fibrosis, including seven residual or recurrent neoplasias after previous resection, and two LST non‐granular pseudo‐depressed (LST‐NGPD) and one LST‐GM in chronic ulcerative colitis.

**Table 1:** Morphological features and histology of 83 large non‐pedunculated colorectal neoplasias referred for endoscopic resection

Morphology of lesions	*n* (%)	Diameter, median (range), mm	Localization rectum *n* (%)	LGIN, *n*	HGIN, *n*	Carcinoma, *n*
Paris type						
0‐ls	10 (12.0)	46 (20–80)	2 (20.0)	6	4	0
0‐lla	29 (34.9)	25 (15–61)	3 (10.3)	27	2	0
0‐lla+ls	27 (32.5)	37 (25–72)	11 (40.7)	14	11	2
0‐lla+llc	15 (18.1)	25 (15–46)	2 (13.3)	7	6	2
0‐lla+llc+ls	2 (2.4)	60 (52–68)	0 (0.0)	1	1	0
LST type						
Granular	42 (50.6)	38 (17–80)	14 (33.3)	24	16	2
Without nodule (LST‐GH)	7 (8.4)	25 (17–54)	2 (28.6)	7	0	0
Mixed type						
Small nodule (<10 mm)	24 (28.9)	42 (25–72)	5 (35.7)	11	12	1
Large nodule (>10 mm)	11 (13.2)	49 (25–80)	7 (63.6)	6	4	1
Non‐granular	41 (49.4)	25 (15–61)	4 (9.8)	31	8	2
Pseudo‐depressed (LST‐NGPD)	15 (18.1)	25 (15–46)	2 (13.3)	7	6	2
Flat/elevated (LST‐NG)	26 (31.3)	24 (15–61)	2 (7.7)	24	2	0
All	83 (100.0)	30 (15–80)	18 (21.7)	55	24	4

HGIN, high‐grade intraepithelial neoplasia; LGIN, low‐grade intraepithelial neoplasia; LST, laterally spreading tumor.

### Technical success and outcome of resections

Endoscopic submucosal dissection intention‐to‐treat was feasible in 96.4% (80/83) of the lesions (*n* = 35 ESD en‐bloc, *n* = 45 H‐ESD), and ended as PM‐EMR in another three (3.6%) cases (i.e. two large LST‐GM [*d* > 60 mm] in the ascending colon, and in the right flexure [both adenomas with LGIN], and one circumferential LST‐GM [*d* = 50 mm; adenoma with focal HGIN] recurrent in the anorectal channel 7 years after transanal endoscopic microsurgery). En‐bloc resection rate was 85%, and increased during the case series (*P* < 0.01, Pearson's chi‐squared test, data not shown). Particularly in lesions with a diameter >40 mm (*n* = 27), the risk for failed en‐bloc resection increased significantly (*P* < 0.01, multivariate analysis). Submucosal fibrosis or scars after previous resection attempts was negatively correlated with en‐bloc resection, irrespective of ESD technique (*P* < 0.01, univariate analysis). R0 resection rate was 74% overall, and correlated with ESD in the standard technique (89%, *P* < 0.01, univariate analysis), and inversely with lesion size (*P* = 0.03, univariate analysis). Data on resection, complications, and outcome are shown in Table 2, statistical analyses in Table 3.

**Table 2:** Endoscopic submucosal dissection (ESD) intention‐to‐treat (ITT) in colorectal large non‐pedunculated neoplasias

	All procedures^a^	ESD	Hybrid‐ESD		Converted to PM‐EMR
All lesions	83 (100)	35 (42.2)	45 (54.2)		3 (3.6)
Localization					
Cecum	14 (16.9)	8 (22.9)	6 (13.3)		0 (0.0)
Right‐sided colon	29 (34.9)	9 (25.7)	18 (40.0)		2 (66.7)
Left‐sided colon	22 (26.5)	5 (14.3)	17 (37.8)		0 (0.0)
Rectum	18 (21.7)	13^d^ (37.1)	4 (8.9)		1 (33.3)
Resection parameters					
Lesion diameter, mm^b^	30 (15–80)	32 (15–72)	25 (15–80)		60^e^ (60–80)
Dissection velocity cm^2^/h^b^	3.8 (0.6–19.7)	5.2^e^ (1.4–19.7)	2.6 (0.6–6.2)		3.8 (1.8–4.5)
Severe sm‐fibrosis	22 (26.5)	5^d^	15		2
Histology					
Carcinoma	4 (4.8)	3 (8.6)	1 (2.2)		0 (0.0)
HGIN	24 (28.9)	11 (31.4)	12 (26.7)		1 (33.3)
LGIN	55 (66.3)	21 (60.0)	32 (71.1)		2 (66.7)
Complications					
Procedure‐associated surgery	1 (1.2)	0 (0.0)	0 (0.0)		1 (33.3)
Stenosis	1 (1.2)	0 (0.0)	0 (0.0)		1 (33.3)
Delayed bleeding	1 (1.2)	0 (0.0)	1 (2.2)		0 (0.0)
Transmural perforation	5 (6.0)	1 (2.9)	4 (8.9)		0^e^ (0.0)
Follow up					
Last follow up, months^b^	24.2 (± 2.4)	18.1 (± 3.3)	27.5 (± 3.2)		17.2 (± 3.4)
*n* lesions	77 (92.8)	31 (88.6)	43 (95.6)		3 (100)
Residual adenoma after R1 resection	4 (4.8)	0 (0.0)	2^e^ (4.4)		2^d^ (66.7)
Recurrence after last follow up^c^	0				
All completed ESD procedures only	80 (96.4)	35 (42.2)	45 (54.2)		
En‐bloc rate	68 (85.0)	35^d^ (100)	33 (73.3)		
R0 rate	59 (73.8)	31^e^ (88.6)	28 (62.2)		
Recurrence after R0 resection	2 (2.4)	0 (0.0)	2 (4.4)		

a ^†^Values are *n* (%), unless otherwise specified.

b ^‡^Median (range) or mean (± SEM).

c ^¶^After endoscopic treatment of recurrent/residual adenomas (by biopsy forceps, argon plasma coagulation or EMR).

d Highly significant (*P* < 0.01, dependent variable on multivariate analysis), detailed statistical analysis is provided in Table 3.

e Significant (*P* < 0.05, dependent variable on univariate analysis), detailed statistical analysis is provided in Table 3.

EMR, endoscopic mucosal resection; ER, endoscopic resection; HGIN, high‐grade intraepithelial neoplasia; LGIN, low‐grade intraepithelial neoplasia; PM‐EMR, piecemeal EMR; sm, submucosal.

**Table 3:** Uni‐ and multivariate analysis: Variables related to outcome

	Predictors	Dependent variables														
		Standard ESD carried out			En‐bloc resection possible			Transmural perforation			R0 resection			Recurrence after R0		
		OR (CI)	Uni‐var.	Multi‐var.	OR (CI)	Uni‐var.	Multi‐var.	OR (CI)	Uni‐var.	Multi‐var.	OR (CI)	Uni‐var.	Multi‐var.	OR (CI)	Uni‐var.	Multi‐var.
Lesion characteristics	Diameter, mm		0.08			0.01	0.41		0.65			0.03	0.32		0.32	
	Diameter >40 mm		0.24		632 (12, 33 668)	**0.00**	**0.00**		0.36			0.02	0.52		0.32	
	Diameter >50 mm		0.48			0.01	0.62		0.48			0.08	0.72		0.45	
	Localization in rectum	0.15 (0.03, 0.70)	**0.00**	**0.02**		0.17			0.19			0.28			0.46	
	Pseudodepressed morphology		0.17			0.24			0.92			0.44			0.50	
Technical variables	Velocity, cm^2^/h				2.76 (1.02, 7.47)	**0.12**	**0.05**		0.22			0.36			0.53	
	Standard ESD carried out				0.00	0.00			0.18			0.00	0.20		0.22	
	En‐bloc resection possible							13.20 (2.13, 82)	**0.00**	**0.01**	0.06 (0.01, 0.25)	**0.00**	**0.00**		0.52	
	Scar after previous ER		0.11			0.06	0.25		0.44			0.97		0.08 (0.00, 1.47)	**0.03**	**0.09**
	Scar after ER and severe sm fibrosis	7.67 (1.7, 33.81)	**0.03**	**0.01**		0.00	0.14		0.71			0.12			0.45	
	PM‐EMR carried out								0.08	0.84		0.77			0.69	
Patient age		0.32			0.31			0.70		0.93 (0.87, 0.99)	**0.02**	**0.04**		0.95		

Binary logistic regression was applied to explore for variables related to technical performance and outcome during ESD implementation. Significant correlations on uni‐ and multivariate analysis are marked in bold.

CI, confidence interval; ER, endoscopic resection; ESD, endoscopic submucosal dissection; OR, odds ratio; PM‐EMR, piecemeal endoscopic mucosal resection.

### Complications

Six (7%) transmural microperforations (one each with ESD, and PM‐EMR, four with H‐ESD) were tightly clipped and remained without clinical consequences, except in one case. A small retroperitoneal perforation in the hepatic flexure was diagnosed and tightly clipped with delay. After 12 h, however, the patient underwent right‐sided hemicolectomy because of rising inflammatory parameters in the presence of retroperitoneal and mediastinal emphysema. All patients were discharged within 10 days after ESD‐ITT. Anorectal stenosis after PM‐EMR was cured with two sessions of balloon dilation. There was no procedure‐related long‐term morbidity or mortality. En‐bloc resection was strongly associated with lower frequency of perforation (*P* < 0.01, multivariate analysis). No other parameters were correlated with perforation on uni‐ and multivariate analysis (Table 3).

### Follow up

Follow up for all patients was mean 24.2 (± 2.4) months, and covered 93% (*n* = 77) of all 83 lesions. Five patients (7.5%) died within median 12 (range 5–13) months unrelated to neoplasia or ESD: two without follow up after R0 resection of adenoma and three with HGIN (*n* = 2) or carcinoma (*n* = 1) without recurrence at first endoscopic follow up after 0.9, 1.4, and 2.9 months, respectively. All 28 (34%) malignant neoplasias were resected R0 with curative criteria, and remained without recurrence at follow up for mean 19.5 (± 3.2) months. Early recurrence after R0 resection was observed in two cases (2.4%) after H‐ESD, associated with submucosal fibrosis from previous resection attempts (*P* = 0.02, univariate analysis). Residual adenoma occurred after R1 resection (*n* = 4; 4.8%)—two each after H‐ESD and PM‐EMR—and was associated with prior submucosal fibrosis (*P* < 0.05, univariate analysis). All recurrent adenomas (*n* = 6) were successfully managed with biopsy forceps removal (*n* = 2), argon plasma coagulation (*n* = 2), EMR (*n* = 1), or endoscopic full‐thickness resection (*n* = 1; FTRD System^®^; Ovesco Endoscopy AG, Tübingen, Germany) without re‐recurrence since mean 19.7 (± 7.2) months.

## Discussion

Data of this prospective case registry show that self‐learning of ESD‐ITT technique is feasible for neoplasias of the entire colorectum with moderate complications (7%) and low need for surgery (1%), high rates of en‐bloc (85%) and R0 resection (74%), curative resection of all (34%) malignant neoplasias, low overall rate of manageable recurrent adenoma (7%), and recurrence‐free long‐term follow up. Major drawbacks are limited sample size and single‐center experience. Nevertheless, this ESD‐ITT approach is consistent with clinical practice in the management of colorectal lesions with indication criteria for ESD^11^ and supports the strategy of “How to implement ESD technique”,^3^, ^6^ provided the data are: (i) not skewed by additional technical training; (ii) unbiased with regard to patients, indications and strategy for ESD; and (iii) the outcome well compares with benchmarks set for ESD.^2^

Each of the three operators had participated in the same experimental ESD training for preparation, including approximately 30 porcine *ex vivo* ESD procedures,^3^ and one annual expert training course on experimental ESD.^7^ Independent self‐learning of ESD was based on principles.^6^ The first operator (F.B.) carried out 50 untutored ESD until he had shown a competence level without complications in 26 consecutive procedures.^6^

Patient characteristics and lesion types and sizes were comparable with other reports (Table 4),^2^ except that we had approached lesions in the entire colon right from the first procedures. Of note, preoperative endoscopic diagnosis (predictive T staging) was accurate, owing to intense cooperation with our expert colleagues from Japan (i.e. clinical LIVE demonstrations and work on book chapters^14^). All lesions indicated for ESD underwent ESD‐ITT. Therefore, the level of technical challenge was high during the initial 25 procedures.^6^ In retrospect, we would not recommend lesions converted to PM‐EMR for learning ESD. However, a referral center for colorectal ESD on a higher professional level was unavailable and these patients preferred ESD to surgery in spite of informed consent on procedure‐ and lesion‐related increased risk of complications.

**Table 4:** Comparison with literature on colorectal endoscopic submucosal dissection (CR‐ESD) (modified from^2^)

Parameter^a^	ESD, standard technique				Hybrid‐ESD			
	Overall	Non‐Asian	Asian	This study	Overall	Non‐Asian	Asian	Present study
*n* studies/patients	97	26	71	35^b^	12	5	7	45^b^
Duration, min	87.7	104.5	82.4	119	57.6	96.0	38.3	141
Lesion size, mm	33.4	37.7	32.3	38.5	31.0	41.0	24.0	29.2
% rectal lesions	46.3	55.2	42.9	37.1	31.6	27.5	34.3	8.9
En‐bloc rate	91.0	81.2	93.0	100.0	68.4	50.1	78.1	73.3
R0 rate	82.9	71.3	85.6	88.6	60.6	44.4	71.1	62.2
Rate of surgery	1.1	3.1	0.8	0.0	1.0	na	na	0.0
Delayed bleeding	2.7	4.2	2.4	0.0	4.0	4.7	3.5	2.2
Perforation rate	5.2	8.6	4.5	2.9	4.8	3.7	5.4	8.9
Recurrence^c^, %	2.0	5.2	1.1	0.0	2.0	na	na	3.6
(95% CI)	(1.3–3.0)	(3.3–8.1)	(0.7–1.8)	na	(0.7–5.6)	na	na	na

a ^†^Values are mean, unless otherwise specified.

b ^‡^ *n* patients.

c ^§^After R0 resection, follow up of 12 months, estimated from univariable meta‐regression models (modified from^2^ with permission from Elsevier).

na, not available.

ESD‐ITT was started with intention for ESD en‐bloc and, when unfeasible, endoscopic resection was completed with snaring. Self‐completion rate was 100% overall: 99% in a single procedure and 1.2% in two sessions. However, we converted ESD to H‐ESD in 54%, and to PM‐EMR in 4% of procedures, mainly for difficult accessibility or/and submucosal fibrosis. Initially, final snaring was often necessary for inexperience with initial complete circumferential incision technique (18% of H‐ESD), and as rescue after complications that had occurred during the ESD approach in the standard technique in four (9%) of the H‐ESD and one PM‐EMR. This reflects clinical practice in the management of colorectal lesions considered as indication for ESD and the associations of failed en‐bloc resection with perforation (Table 3), although none of the snaring maneuvers had been complicated by perforation. In Japan, trainees during their first 40–45 colorectal ESD procedures had achieved complete endoscopic resection either with final snaring in 13–39% or as completion by the tutor in 20–35% of procedures.^22^, ^24^, ^25^ In the West, trainees used final snaring (H‐ESD or PM‐EMR) for self‐completion.^6^, ^26^, ^27^, ^28^ Considering that we had included all indications, even those requiring a high level of skill, the 54% rate of H‐ESD appears equivalent to combined rates of H‐ESD and non‐self‐completion reported for trainees from major centers in Japan.^22^, ^24^, ^25^

The perforation rate of 7% with ESD‐ITT well compares with 4–12.5% perforations during supervised learning of colorectal ESD.^22^, ^24^, ^25^ Our perforation rate with pooled analysis of ESD and H‐ESD corresponds to the benchmark in Asia (Table 4), and was lower (2.3%) during the second 43 procedures. Delayed bleeding, and need for surgery (1.2% each) were at benchmark level. Thus, colorectal ESD‐ITT was a safe approach.

Pooled analysis (*n* = 80) of standard ESD and H‐ESD achieved en‐bloc resection in 85% of ESD procedures, and R0 resection in 74%. Of note, all malignant neoplasias (34%) had been R0 resected, and none had high‐risk criteria or need for surgery. Uncomplicated H‐ESD for self‐completion showed 4.4% (2/45) adenoma recurrence, much lower than the 14% recurrence after a priori PM‐EMR.^2^, ^29^ Manageable recurrent adenoma was observed in 7% after ESD‐ITT—including two cases (2.4%) after R0 resection (both H‐ESD with severe fibrosis), and four (4.8%) after R1 resection (2 H‐ESD for perforation; 2 PM‐EMR)—and all remained in remission after endoscopic ablation, yielding zero rate of local recurrence in the long term.

In conclusion, the main findings for independent implementation of ESD technique in the entire colorectum were: (i) accurate endoscopic indication and curative resection of malignant lesions is feasible; (ii) ESD‐ITT in the entire colorectum shows a low recurrence rate; and (iii) hybrid‐ESD is a self‐completion and rescue strategy with acceptable complication and recurrence rates.

## Conflicts of Interest

Authors declare no conflicts of interest for this article.
